# Adult‐onset epidermal nevus with epidermolytic hyperkeratotic pattern: Case report and dermoscopic findings

**DOI:** 10.1002/ccr3.3144

**Published:** 2020-07-20

**Authors:** Ansari Mahshid Sadat, Nasimi Maryam, Kamyab Kambiz, Mahmoudi Hamidreza

**Affiliations:** ^1^ Department of Dermatology Razi Hospital Tehran University of Medical Sciences Tehran Iran; ^2^ Department of Dermatopathology Razi Hospital Tehran University of Medical Sciences Tehran Iran

**Keywords:** dermoscopy, epidermal nevus, epidermolytic hyperkeratosis

## Abstract

EN is a hamartomatous proliferation of keratinocytes. The most common dermoscopic feature of EN is large brown circles in the absence of pigment network which is similar in different histopathological variants including epidermolytic hyperkeratotic.

## Graphical abstract

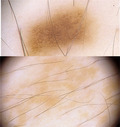

## 1 INTRODUCTION

Epidermolytic hyperkeratotic EN (epidermal nevus) is a rare variant of EN, which can be congenital or developed later in adulthood. A 32 years old female presented with a pathologic confirmed epidermolytic hyperkeratotic EN. Dermoscopy can be helpful in the diagnosis of EN, but not in differentiating the variant.

Epidermal nevus is a hamartomatous proliferation of keratinocytes. It usually appears during the first year of life and gradually increases in thickness, although some cases of late‐onset development have also been reported.^1^ Epidermal nevus is usually an isolated benign lesion, but especially when it is extensive, it may be associated with other organ and system abnormalities which is called epidermal nevus syndrome.^2^

Dermoscopy is a noninvasive diagnostic method that can be used readily to differentiate melanocytic and nonmelanocytic lesions. It also helps in biopsy decision‐making for a suspected lesion.^2^, ^3^

Herein, we report an acquired case of EN in a 32‐year‐old woman with epidermolytic hyperkeratotic pattern and present a review of the literature about it.

## 2 CASE PRESENTATION

**FIGURE 1:**
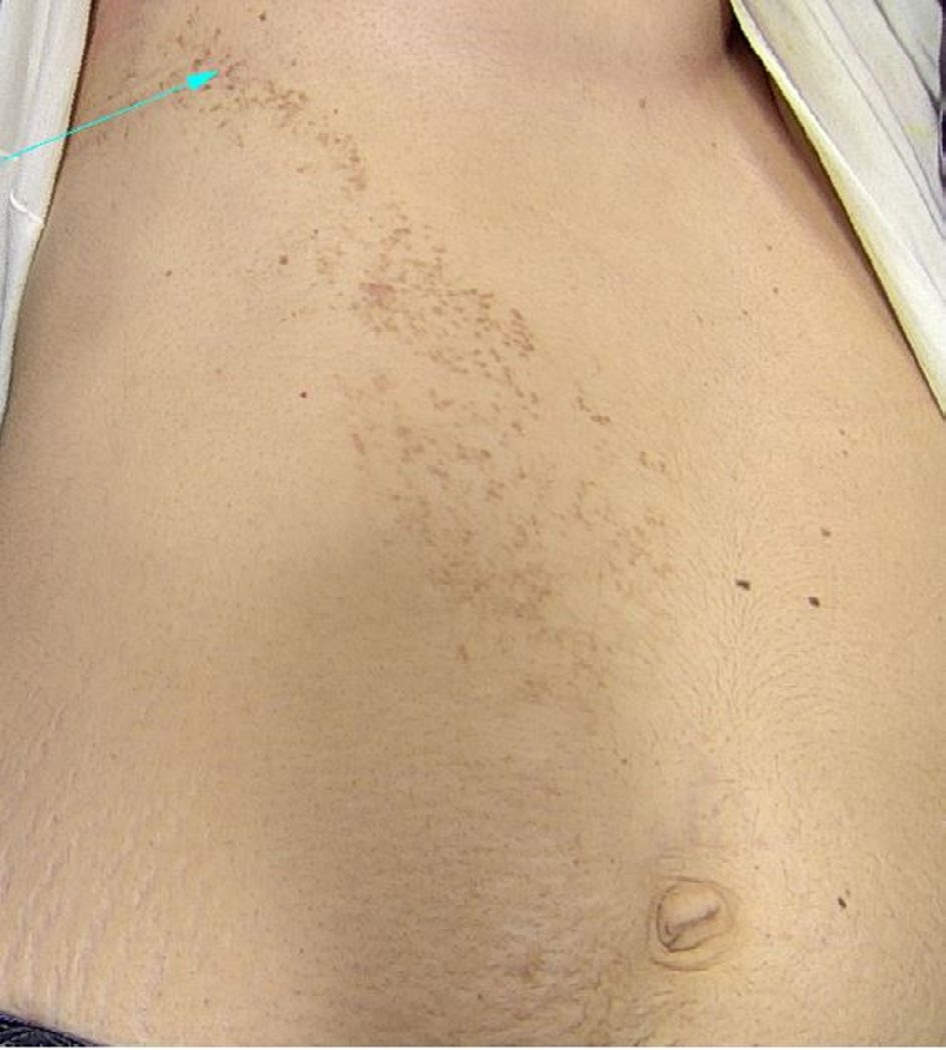
Unilateral brown papules along blaschko's lines

A 32‐year‐old woman presented with a unilateral small brown blaschkoid sessile and pedunculated papules on the right side of her abdomen in the past 4 years (Figure 1). She did not complain from any symptom except for occasional pruritus. The lesions appeared during her first pregnancy and became more prominent and increased in number during her second pregnancy. Past medical history and examination were otherwise unremarkable.

**FIGURE 2:**
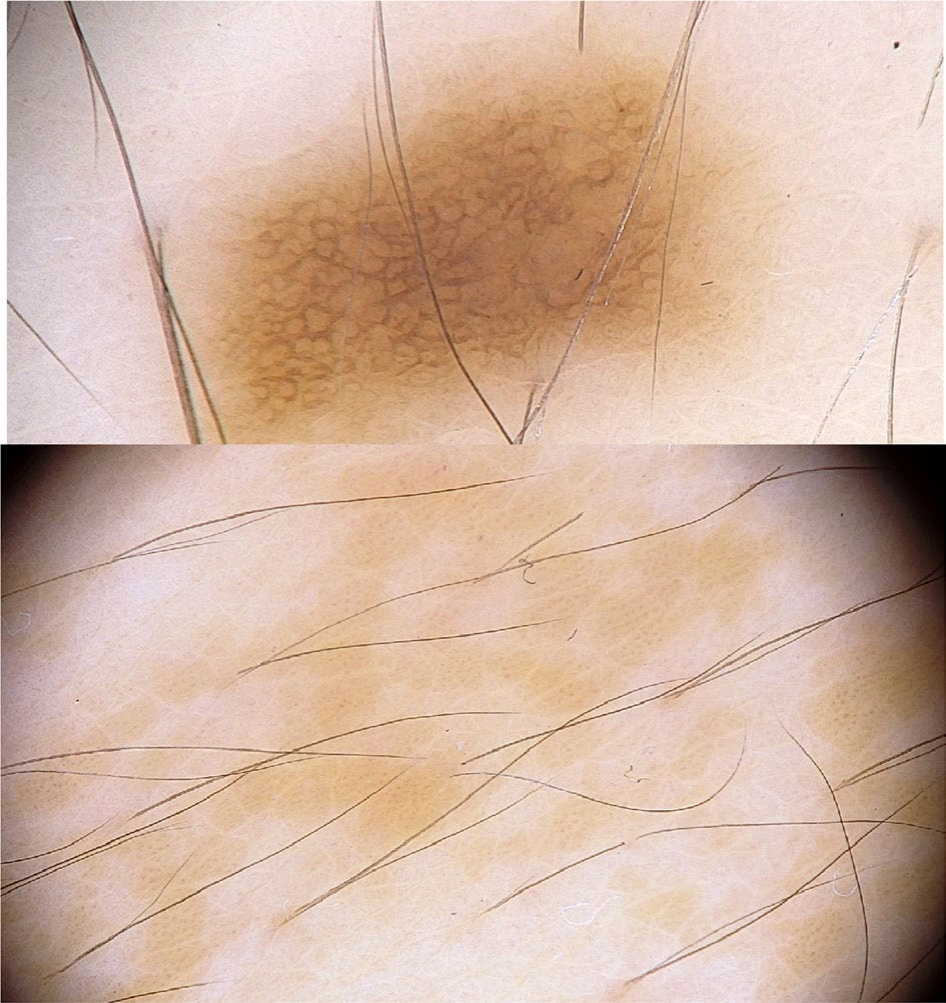
Dermoscopic examination: large and small brown circles in pigmented background

On dermoscopic examination, multiple round and oval brown pigmented circles with irregular borders and some smaller circles were seen in a homogenous light‐brown background (Figure 2).

**FIGURE 3:**
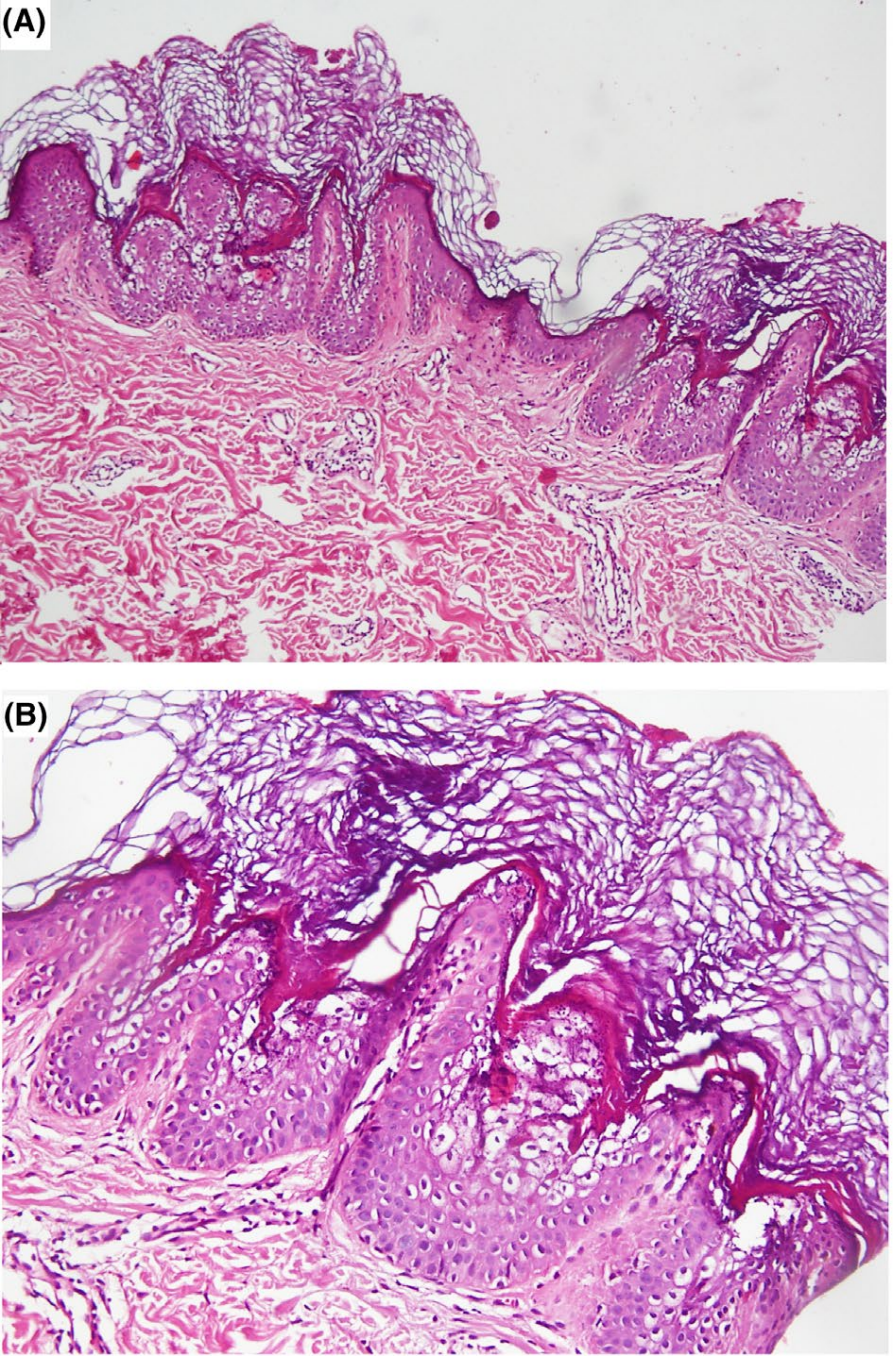
A, Section shows epidermis with elongated rete ridge and orthokeratotic hyperkeratosis (H&E, magnification ×10). B, High magnification reveals vacuolated cells. (H&E, magnification ×40)

Histology examination revealed a thick orthokeratosis, papillomatosis, coarse keratohyalin granules, and some vacuolated cells with dark basophilic nuclei known as epidermolytic hyperkeratotic pattern. Some superficial lymphocytic infiltration was also noted in the dermis. Regarding the history, clinical manifestation and histopathological findings diagnosis of epidermolytic hyperkeratotic EN was made (Figure 3).

## 3 DISCUSSION

Epidermal nevus is fundamentally a hamartoma of keratinocytes and occasionally adnexal structures. It mostly develops in the first years of life, although late‐onset presentation has also been reported.^1^, ^4^ Martinez et al report a 27 years old woman who developed EN during pregnancy. They proposed hormonal and immunological alternations during pregnancy as a possible explanation.^5^

More than 10 histopathological variants of EN have been described so far, and papillomatosis, hyperkeratosis, and acanthosis are the most common findings. Epidermolytic hyperkeratosis is an uncommon variant.^6^

Epidermolytic hyperkeratosis is characterized by granular and vacuolar degeneration of keratinocytes in the spinous and granular layer of epidermis along with hyperkeratosis. In addition to EN, this histological pattern can be seen in epidermolytic ichthyosis, specific kinds of palmoplantar keratodermas and epidermolytic acanthoma.^7^

The dermoscopy view of the epidermolytic hyperkeratosis EN is mostly similar to other kinds of EN containing: large brown circles and the absence of the pigment network.^2^, ^8^ Carbotti et al first described large brown circles in EN and correlated it histologically to the way pigmented keratinocytes lie down around dermal papillae.^2^ Demographics and dermoscopic features of epidermolytic hyperkeratosis EN being reported in the literature are summarized in Table 1.

**Table 1:** Dermoscopic features in previous studies

	Carbotti et al^2^ (N = 8)	Verzi et al^8^ (N = 9)	Elmas et al^12^ (N = 20)	Arjona‐Aguilera et al^13^ ^b^ (N = 1)	Current study (N = 1)
Sex	F = 6/M = 2	–	F = 9/M = 11	M	F
Median age (y)	38	–	24	10	32
Location					
Trunk	5/8	–	7/20	−	+(Abdomen)
Face	–	–	9/20	−	−
Neck	1/8	–	4/20	+	−
Extremities	2/8	–	–	−	−
Histopathology	VEN^a^	VEN	VEN	Acanthosis nigricans form	Epidermolytic hyperkeratosis
Dermoscopic findings					
Large brown circles	8/8	9/9	10/20	−	+
Comedo‐like opening	3/8	–	7/20	−	−
Cerebriform pattern	–	–	1/20	+	−
Thick brown line	–	–	9/20	−	−
Globule	–	–	+(brown)	+(black)	−
Small brown circles	–	–	–	−	+
Dotted vessels	–	–	7/20	−	−

^a^ Verrucous epidermal nevus is defined by hyperkeratosis, papillomatosis, acanthosis, and elongation of rete ridges.

^b^ Other more frequent findings were whitish and brown exophytic papillary structures, terminal hair, and scale.

The clinical differential diagnosis of this lesion includes wart and seborrheic keratosis which can be excluded by considering the unique clinical pattern of EN (blaschkoid distribution) and lack of cytopathic viral effects, which are seen in the wart. Dermoscopy can be used to differentiate these lesions. Regularly distributed red dots in the yellow to light‐brown background can be demonstrated in wart and milia‐like cyst, comedo‐like openings, network‐like structures, cerebriform pattern and hairpin blood vessels are the most frequent findings in seborreic keratosis.^9^, ^10^

Full‐thickness surgical excision is the treatment, but in large lesions, hypertrophic scar and keloid formation is the side effect. Other treatment modalities include electrofulguration, cryosurgery, and dermabrasion, but they make scarring. Topical agents (calcipotriol, steroids, retinoic acid) were not effective. Soft and flat nevi were responsive to ablative lasers, but the lesions may recur after nonsurgical procedures. So because of the side effects of extensive surgery, a conservative approach is logic in the case of extensive lesions.^11^

In conclusion, epidermolytic hyperkeratotic EN is a rare variant of EN, which can be congenital or developed later in adulthood. Although the histopathological finding of this variant is unique and is different from other variants of EN, the dermoscopic feature is the same and can be considered an important clue in the diagnosis of different variants of EN.

## CONFLICT OF INTEREST

None declared.

## AUTHOR CONTRIBUTIONS

Mahshid Sadat Ansari and Kambiz Kamyab: involved in acquisition of data, or analysis and interpretation of data. Maryam Nasimi: drafted the manuscript or revised it critically for important intellectual content. Hamidreza Mahmoudi: conceptualized and designed the study, and gave final approval of the version to be published.

## ETHICAL APPROVAL

The study was approved by the ethics committee, and consent of patient was obtained.
